# Abdominal bleeding secondary to isolated mesenteric injury following blunt abdominal trauma: A case report

**DOI:** 10.1097/MD.0000000000041920

**Published:** 2025-03-21

**Authors:** Zhiqiang Ming, Chao Xiao, Rui Xiao, Yongtao Zhang, Xiaoli Hu

**Affiliations:** a Department of Radiology, Zigong First People’s Hospital, Zigong, Sichuan Province, China; b Department of Obstetrics and Gynecology, Zigong First People’s Hospital, Zigong, Sichuan Province, China; c Department of Obstetrics and Gynecology, West China Second University Hospital, Sichuan University, Chengdu, China; d Department of Ultrasound, Zigong First People’s Hospital, Zigong, Sichuan Province, China.

**Keywords:** abdominal bleeding, computed tomography, isolated mesenteric injury

## Abstract

**Rationale:**

Isolated mesenteric injury often results from traffic accidents causing blunt abdominal trauma, exhibit nonspecific symptoms and signs. Most mesenteric injuries occur concurrently with injuries to other organs, such as the liver and spleen. As a result, the incidence of isolated mesenteric injury is very low and often misdiagnosed.

**Patient concerns:**

A 49-year-old male patient presented with abdominal pain following a traffic accident more than 5 hours before admission. Upon admission, his blood pressure was normal but dropped sharply in a short time and presented with shock. Anemia, abrasions in the upper abdomen, cold skin on the dorsum of the foot, rebound pain, muscle tension, and noncoagulation of abdominal blood were all observed during the physical examination. Emergency plain and contrast-enhanced abdominal computed tomography (CT), and mesenteric artery CT angiography revealed a large volume of blood accumulated in the abdomen, pelvis, and jejunal mesentery. The jejunal wall and mesentery were edematous, with mesenteric distortion, and some branches of the inner jejunal artery were not clearly visualized. We found no evidence of liver or splenic rupture.

**Diagnoses:**

Mesenteric injury of the jejunum caused acute hemorrhagic anemia and hemorrhagic shock.

**Interventions:**

An emergency laparotomy was performed.

**Outcomes:**

An emergency laparotomy showed 3 hematomas close to the perforation site, 2 fully split tears in the jejunal mesentery, bleeding from partially exposed veins, a significant amount of blood and clots in the abdominal cavity, and serosal damage at the jejunal mesentery’s root. The patient recovered well after we performed abdominal cavity drainage and jejunal mesenteric hemostasis and repair.

**Lessons:**

Isolated mesenteric injuries are very rare in clinical practice, and their clinical symptoms and signs are nonspecific, which makes them prone to misdiagnosis and oversight. When a patient satisfies the 4 requirements listed below: abdominal hemorrhage or hematoma; a history of abdominal trauma; no damage to high-risk organs like the liver or spleen; the CT-detected signs of mesenteric injury. Abdominal paracentesis or laparoscopy should be conducted to confirm the diagnosis and initiate further treatment.

## 1. Introduction

Even though mesenteric injury occurs in only 1.0% to 5.0% of blunt abdominal trauma cases, and even fewer in isolated cases, the mortality rate is substantial in these cases. Mesenteric injuries often result from traffic accidents causing blunt abdominal trauma,^[1–3]^ exhibit nonspecific symptoms and signs, and occur concurrently with injuries to other organs, such as the liver and spleen. Due to inertial thinking, mesenteric injuries are often misdiagnosed, leading to delayed treatment.^[4]^

## 2. Case presentation

**Figure 1.**
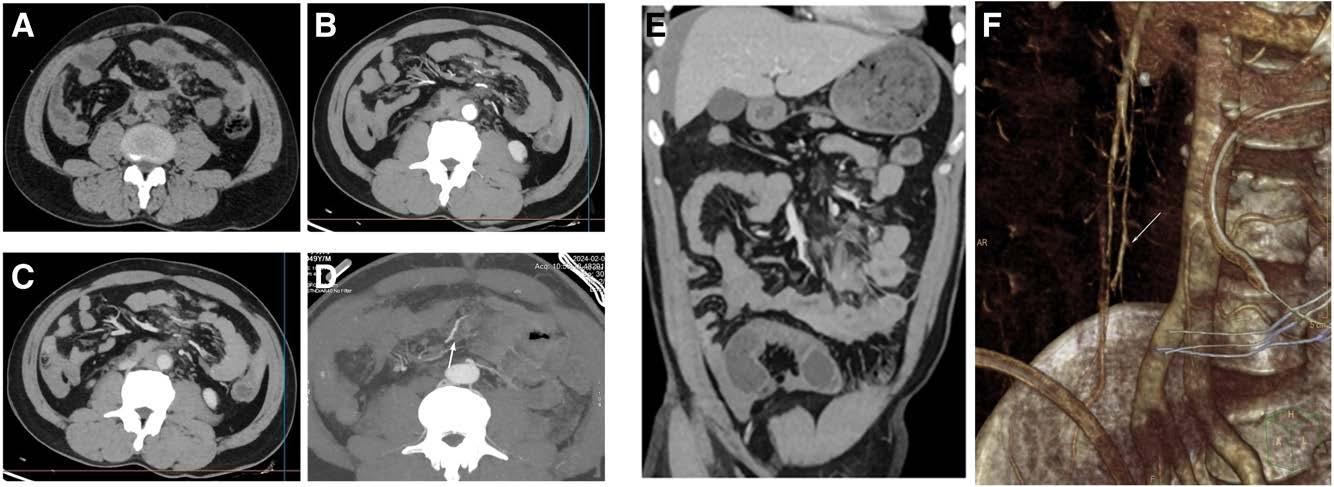
(A–C) Axial CT plain scan, enhanced CT arterial phase, and portal vein phase, respectively. (D) The maximum intensity projection of mesenteric artery CTA. (E) The portal phase of coronal CT enhancement. (F) Mesenteric artery CTA volume reconstruction. CT = computed tomography, CTA = computed tomography angiography.

A 49-year-old man presented with abdominal pain following a traffic accident that occurred more than 5 hours prior to admission. Emergency plain and contrast-enhanced abdominal computed tomography (CT), along with mesenteric artery CT angiography, revealed a large volume of blood accumulated in the abdomen, pelvis, and jejunal mesentery. The jejunal wall and mesentery appeared edematous, with mesenteric distortion, and some branches of the inner jejunal artery were not clearly visualized. The CT images showed no signs of liver or splenic rupture, based on these findings, the radiology department diagnosed the patient with jejunal mesenteric injury (Fig. 1).

Upon admission, his blood pressure was normal but dropped sharply in 3 hours and presented with shock. Anemic appearance, abrasions in the upper abdomen, cold skin on the dorsum of the foot, rebound pain, and muscle tension were all observed during the physical examination. Non-coagulating blood was acquired by abdominal puncture.

The patient was diagnosed with acute hemorrhagic anemia and hemorrhagic shock. An emergency exploratory laparotomy revealed a large volume of blood and clots in the abdominal cavity, totaling approximately 800 mL. There were 2 completely split pores in the jejunal mesentery, measuring approximately 10 cm × 7 cm and 8 cm × 5 cm, with some blood vessels exposed and showing hemorrhage. Near the hiatus, there were 3 hematomas, measuring 5 cm × 4 cm, 4 cm × 4 cm, and 4 cm × 3 cm, respectively. Additionally, rupture of the mesentery and serosa, rupture of the serosa at the root of the small bowel mesentery, and exposure of some blood vessels were observed.

The blood in the abdominal cavity was suctioned out, and the blood clots were removed. Due to the extensive serosal damage at the root of the small mesentery, which could not be repaired, and the exposure of internal blood vessels, the adjacent omentum was taken and attached to the serosal damage surface, then sutured and fixed. Two units of red blood cells were transfused before the surgery. The patient showed good postoperative recovery, and checked out in 4 days.

## 3. Discussion and conclusions

A literature review was conducted in PubMed using the search terms “blunt abdominal trauma” and “mesenteric injury,” with the scope limited to [Title/Abstract]. This search yielded 81 articles. We read the abstracts of these 81 articles and found that 10 of them involved “isolated mesenteric injury.” We further read the full texts of these articles and analyzed the data of 15 cases involving isolated mesenteric injury. We summarized age, gender, reporting time, main symptoms, causes of injury, mesenteric injury type, secondary changes, main diagnostic methods, and treatment methods, with the specific content shown in Table 1.^[5–14]^

**Table 1:** Case collection of isolated mesenteric injury due to traumatic blunt abdominal injury.

No	Age	Sex	Year	Symptoms and signs	Causes of injury	Mesenteric injury type	Secondary changes	Main diagnostic methods	Treatment	Reference
1	30	Male	2020	Abdominal pain	Car accident	Small intestinal mesentery and superior mesenteric artery injury	NA	Enhanced CT	Surgery	^[5]^
2	31	Female	2020	Abdominal pain	Car accident	Injury to the mesentery of the ileum	Small bowel perforation	Enhanced CT	Surgery	^[5]^
3	52	Male	2020	Abdominal pain	Car accident	Perforation of the mesentery	Bowel perforation	Enhanced CT	Surgery	^[5]^
4	51	Male	2020	Abdominal pain	Car accident	Perforation of the jejunum mesentery	Small bowel perforation	Enhanced CT	Surgery	^[5]^
5	55	Female	2020	Abdominal pain	Car accident	Injury to the mesentery	NA	Enhanced CT	Surgery	^[5]^
6	68	Male	2020	Abdominal pain, shock	Abdominal massage	Laceration of the transverse mesocolon	NA	Enhanced CT, abdominal puncture	Surgery	^[6]^
7	75	Male	2024	Worsening abdominal pain	Tennis racket handle hit the abdomen	Mesenteric injury combined with mesenteric artery injury	NA	Enhanced CT	Nonsurgical	^[7]^
8	39	Male	1976	Abdominal tenderness	Car accident	Transverse mesentery injury, the middle colic artery has been cut off	Intestinal necrosis	Enhanced CT	Surgery, the necrotic bowel was resected	^[8]^
9	39	Male	1976	Abdominal tenderness	Car accident	There were 2 tears in the mesentery of the ileum	NA	Enhanced CT	Part of the bowel was surgically removed	^[8]^
10	51	Male	2015	Abdominal pain, constipation, and vomiting	Car accident	Omentum injury	Small bowel torsion, obstruction, necrosis	Enhanced CT, ultrasound, exploratory laparotomy	The diseased bowel was resected surgically	^[9]^
11	54	Male	2016	Abdominal pain, diffuse tenderness	He fell and injured his abdomen	Small mesenteric hematoma	Mesenteric adhesions, small bowel obstruction	Enhanced CT	Part of the small intestine was surgically removed	^[10]^
12	32	Male	2016	Abdominal pain	Motorcycle accident	Mesenteric hematoma	Small bowel perforation	Enhanced CT, exploratory laparotomy	Surgery	^[11]^
13	33	Male	2017	Abdominal pain	Car accident	Injury to the mesentery	The small intestine was necrotic and perforated	CT, exploratory laparotomy	Surgery	^[12]^
14	32	Male	2020	Nausea, Vomiting	Car accident	Hematoma of the colon	Intestinal obstruction	CT, ultrasound, exploratory laparotomy	Part of the bowel was surgically removed	^[13]^
15	21	Male	2022	Severe colic in the right hypochondriac region	A blow to the abdomen while playing basketball	Mesenteric hematoma	NA	CT, ultrasound	Nonsurgical	^[14]^
16	49	Male	2024	Abdominal pain	Car accident	Mesenteric and vascular injuries in the small intestine	NA	Enhanced CT, exploratory laparotomy	Surgery	Present case

CT = computed tomography, NA = not applicable.

Only 15 cases of blunt abdominal injury involving isolated mesenteric injury were identified, indicating that isolated mesenteric injury is relatively rare. Their ages ranged from 21 to 75. Out of 16 cases, 11 were due to traffic accidents, and 2 involved females. The most common type of mesenteric injury was to the small intestinal mesentery, often resulting in necrosis and perforation in delayed treatment cases. Enhanced CT and the exploratory laparotomy are the main method for diagnosis.

Most of the cases presented with abdominal pain or tenderness, and 1 case presented with shock, which indicated that the clinical symptoms and signs of isolated mesenteric injury were nonspecific. In some cases, abdominal pain and other symptoms continue to worsen, potentially secondary to intestinal necrosis and other serious complications. In addition, most mesenteric injuries are often associated with damage to the liver, spleen, and other organs. If there is a definite intra-abdominal bleeding, clinicians, influenced by their inertial experience, typically focus a great deal on damage to high-risk organs to locate the bleeding site, often ignoring mesenteric injuries.

Among the 16 cases listed in Table 1, only 2 were treated with non-surgical methods, while the rest underwent surgical treatment. In some cases, part of the bowel was removed, indicating that surgery is the primary treatment for mesenteric injury. Mesenteric lacerations, particularly those at the base of the mesentery where larger blood vessels are located, can cause heavy bleeding and rapidly lead to shock. Patients should receive concurrent anti-shock treatment and undergo an emergency laparotomy. The timing of the operation, which should be as soon as possible, is closely related to the prognosis.^[15,16]^ For isolated mesenteric lacerations with a good blood supply, perform hemostasis only, avoiding the blind clamping of mesenteric blood vessels to prevent aggravating mesenteric injury that could affect blood supply. When mesenteric hematoma thrombosis leads to intestinal necrosis, 1 must decisively perform resection and anastomosis to prevent serious consequences. In cases of mesenteric injury with bowel necrosis or perforation that results in pelvic contamination, intestinal colostomy surgery is warranted for the patient’s safety.

CT serves as an important preoperative examination method for patients with mesenteric injuries. We must recognize its significant radiological manifestations: Hemorrhage or hematoma in the abdominal cavity, especially in the mesenteric area, where free blood often takes on a triangular shape with the apex pointing towards the root of the mesentery; swelling of the mesentery and disarray of its structure; or clear mesenteric vascular injuries, such as sudden interruption or irregularity of blood vessels or extravasation of contrast agent. Corresponding segments of the intestine become swollen due to ischemia and may even undergo necrosis, perforation, and peritonitis. When a patient meets at least one of the above conditions, combined with a history of abdominal trauma and symptoms such as abdominal pain, peritonitis, or even shock, we should suspect the presence of mesenteric injury.

In conclusion, isolated mesenteric injuries are very rare in clinical practice, and their clinical symptoms and signs are nonspecific, which makes them prone to misdiagnosis and oversight. When a patient satisfies the 4 requirements listed below: Abdominal hemorrhage or hematoma; A history of abdominal trauma; No damage to high-risk organs like the liver or spleen; The CT-detected signs of mesenteric injury that we previously discussed. Abdominal paracentesis or laparoscopy should be conducted to confirm the diagnosis and initiate further treatment.

## Author contributions

**Conceptualization:** Zhiqiang Ming, Xiaoli Hu, Chao Xiao.

**Data curation:** Zhiqiang Ming, Xiaoli Hu, Rui Xiao, Yongtao Zhang.

**Project administration:** Xiaoli Hu.

**Resources:** Rui Xiao, Yongtao Zhang, Chao Xiao.

**Supervision:** Zhiqiang Ming, Xiaoli Hu.

**Validation:** Zhiqiang Ming.

**Visualization:** Zhiqiang Ming.

**Writing – original draft:** Zhiqiang Ming, Xiaoli Hu.

**Writing – review & editing:** Zhiqiang Ming, Xiaoli Hu, Rui Xiao, Yongtao Zhang, Chao Xiao.
